# A critical insight on nanofluids for heat transfer enhancement

**DOI:** 10.1038/s41598-023-42489-0

**Published:** 2023-09-15

**Authors:** Abdul Hai Alami, Mohamad Ramadan, Muhammad Tawalbeh, Salah Haridy, Shamma Al Abdulla, Haya Aljaghoub, Mohamad Ayoub, Adnan Alashkar, Mohammad Ali Abdelkareem, Abdul Ghani Olabi

**Affiliations:** 1 Sustainable and Renewable Energy Engineering Department, University of Sharjah, P.O. Box 27272, Sharjah, United Arab Emirates; 2 Sustainable Energy & Power Systems Research Centre, RISE, University of Sharjah, P.O. Box 27272, Sharjah, United Arab Emirates; 3 School of Engineering, Lebanese International University LIU, Mazraa, P.O. Box: 146404, Beirut, Lebanon; 4 School of Engineering, International University of Beirut BIU, Mazraa, P.O. Box: 146404, Beirut, Lebanon; 5 Industrial Engineering and Engineering Management Department, University of Sharjah, P.O. Box 27272, Sharjah, United Arab Emirates; 6 Benha Faculty of Engineering, Benha University, P.O. Box 13511, Benha, Egypt; 7 Materials Science and Engineering PhD Program, American University of Sharjah, P.O. Box 26666, Sharjah, United Arab Emirates

**Keywords:** Materials science, Nanoscale materials, Mechanical engineering

## Abstract

There are numerous reports and publications in reputable scientific and engineering journals that attribute substantial enhancement in heat transfer capabilities for heat exchangers once they employ nanofluids as working fluids. By definition, a nanofluid is a working fluid that has a small volume fraction (5% or less) of a solid particle with dimensions in the nanoscale. The addition of this solid material has a reported significant impact on convective heat transfer in heat exchangers. This work investigates the significance of the reported enhancements in many recent related publications. Observations on these publications’ geographical origins, fundamental heat transfer calculations, experimental setups and lack of potential applications are critically made. Heat transfer calculations based on methodologies outlined in random selection of available papers were conducted along with a statistical analysis show paradoxically inconsistent conclusion as well as an apparent lack of complete comprehension of convective heat transfer mechanism. In some of the surveyed literature for example, heat transfer coefficient enhancements were reported to be up to 27% and 48%, whereas the recalculations presented in this work restrain proclaimed enactments to ~ 3.5% and − 4% (no enhancement), respectively. This work aims at allowing a healthy scientific debate on whether nanofluids are the sole answer to enhancing convective heat transfer in heat exchangers. The quantity of literature that confirms the latter statement have an undeniable critical mass, but this volition could be stemming from and heading to the wrong direction. Finally, the challenges imposed by the physical nature of nanoparticles, as well as economic limitations caused by the high price of conventional nanoparticles such as gold (80$/g), diamond (35$/g), and silver (6$/g) that hinder their commercialization, are presented.

## Introduction

There are many scientific points of contention in the presentation of results for the claimed enhancement in either the working fluid heat transfer properties (thermophysical) or in the alleged enhancement of heat transfer in general. This fact is found to be true for the majority of the articles reviewed in this work, where the definition and calculation of heat transfer and its desired departure from the baseline working fluid performance is either not clear or completely erroneous.

It is well-known that convective heat transfer is one of the most complex phenomena in heat transfer. Physically, it commences as conduction between a solid surface and adjacent static fluid molecules, which is so far from a pure conduction problem and heat transfer can be determined accurately and easily. But as the molecules gain (or lose) thermal energy, their specific volume also changes accordingly, and a relative motion is initiated by the thermally induced density gradient like hot air balloons. This motion capitalizes on the intrinsically generated buoyancy force. As fluid motion gains more momentum (signified by the velocity increase of particles), the mechanism of heat transfer switches to convection, as the conditions for conduction are no longer applicable and its contribution diminishes.

Heat transfer by convection is a complex phenomenon, as it is directly influenced by several interdependent factors that relate not only to fluid type or properties but also to the nature of fluid flow. It is important to mention a trivial fact that may be inconspicuous for students and researchers of convective heat transfer. This mode of heat transfer is unimportant if it is not in direct contact with a solid surface at a temperature different than that of the working fluid. Anything far enough from the solid surface (or body) is free from thermal and viscous effects that arise due to the proximity of the fluid to that solid. The geometrical shape, surface roughness and physical boundaries of the solid play a significant role in the quality of heat transfer within the fluid as it is responsible for mixing vigor and thus the homogeneity of the heat transfer. The thermophysical properties of the fluid, on the other hand, are also responsible for the quality of heat transfer between the fluid molecules. The thermal conductivity, heat capacity and density are all factors that directly affect heat transfer and it is desirable to couple the fluid flow parameters and thermophysical properties of the fluid in such a manner as to achieve an as high as possible heat transfer effectiveness.

This work discusses the limitations and implications of conventional heat transfer coefficient calculation methods. A summary on previously conducted literature review is shown in Table 1. They provide insights on the preparation of nanofluids, enhancements as proclaimed by corresponding researchers, as well as the utilization of nanofluids across different applications. However, they seem to lack perspective on the calculations carried out by the reported research, which may give a false sense of enhancement in some cases. As the conducted literature review reveals that only a small percentage of authors have thoroughly assessed the convective heat transfer problem from both fluid flow and thermophysical properties perspectives. Discretizing problems, leading to measuring the thermophysical properties of nanomaterials and applying deterministic heat transfer equations, without taking into account the biases introduced by this approach, is a common ground between a myriad of published works. Moreover, experimental setups for heat transfer measurement lack sensitivity and error analysis. Which is why this work puts into perspective the recalculation of the convective heat transfer coefficients from previously published work, utilizing suitable convective heat transfer relations to examine the proclaimed enhancements. Furthermore, the paper examines maintenance and implementation challenges of using nanofluids in practical heat exchangers, including agglomeration, impaired heat transfer, and the high cost of conventional nanoparticles.

**Table 1:** The scope of previously conducted literature reviews on nanofluids research.

Scope	Ref
Studies the preparation of hybrid nanofluids as well as reports of enhancement, stability and measurement techniques	^1^
Studies the application of nanofluids in various heat transfer systems	^2^
Studies the adverse effects on nanofluid stability including the Zeta potential, pH value, and nanoparticle microstructure	^3^
Studies the compatibility of hybrid and mono nanofluids with different heat transfer applications	^4^
Compares between various heat transfer systems with similar Reynolds number conditions, utilizing nanofluids under laminar and turbulent regimes	^5^

## Convective heat transfer basics and enhancements

Analyzing the interaction of the two complex sets of influential parameters (fluid flow and thermophysical) has been the holy grail of any research in convective heat transfer. Fluid dynamics is currently governed by the relationships established by Navier–Stokes and documented by their equations. In these equations, the effects of interaction of body, pressure, and viscous forces on the change of velocity in both magnitude and direction in all three dimensions is detailed. These famous equations sets still cannot be analytically solved and thus cannot be applied to scenarios under laboratory control, let alone in real life applications.

What Bernoulli was able to achieve in simplifying the Navier–Stokes equation for one dimensional, incompressible, and inviscid flow (a single line, simple equation) has resulted in all domestic, industrial, military and aerospace advancements relating to fluid flow to date. Of course, his equations needed modifiers in the form of friction graphs (Moody diagram) or compressible flow assumptions (polytropic flow around shockwaves).

As for heat transfer analysis, even with the rare situations where the thermophysical properties are unambiguously defined, once the convective mechanism becomes the most dominant heat transfer mode the estimation of energy transfer can be too divergent from real or measured values. The relative simplicity of Newton’s law of cooling, which is the following equation:

1 $$Q=hA({T}_{s}-{T}_{\infty })$$

where *Q* is the heat transfer in [J], *A* is the solid surface area in contact with the fluid in [m^2^], ${T}_{s}$ and ${T}_{\infty }$ are surface and ambient temperatures in [K], respectively.

The convective heat transfer coefficient, *h* in [J/m^2^K], seems deceptively easy to determine from this straightforward relationship. But if one is to examine its values from any heat transfer textbook, its variation can be from 2 J/m^2^K for natural convection of air up to 100,000 J/m^2^K for turbulent flow heat transfer that involves phase change of a liquid working fluid. This is an indication of the complexity of the heat transfer problem under convection. The accuracy in determining the convective heat transfer coefficient so it can be plugged into Eq. (1) proves to be an unsurmountable impasse for any practical application, mainly due to the lack of direct coupling of heat transfer with the influence of fluid flow.

By consulting most practical heat transfer textbooks and speaking from a pragmatic engineering perspective, empirical formulas were heavily used by engineers and practitioners to facilitate the speed with which they obtained answers to heat transfer problems. All empirical formulas are practical and save extensive (and precious) time needed to setup and solve analytical or simulation models. The empirical models are currently properly documented in literature and are taught in most engineering heat transfer courses. The main and obvious drawback of employing empirical formulas in heat transfer is that they all come with a disclaimer that if their boundary conditions are not obeyed or experimental constraints observed, the results can be off by as much as 30% in some cases. Still, for most practical applications, this percentage is a small price to pay if a solution is offered in a fraction of the time needed for simulation and with a clear error margin that can be incorporated into a factor of safety that has a finite and measurable effect on the price of the heat transfer solution being designed.

## Experimental assessment of convective heat transfer

Convective heat transfer phenomenon is quite complex, and its mathematical analysis requires rigorous solution of multi-variate and multi-dimensional system of differential equations. One pathway to follow the empirical router and feed simplified forms of these differential equations with well-designed experimental results. The empirical formulae used to assess convective heat transfer take the following general form:

2 $$\mathrm{Nu}=\frac{hD}{k}=C{\mathrm{Re}}^{m}{\mathrm{Pr}}^{n}$$

where Nu is the dimensionless Nusselt number, *h* is the convective heat transfer coefficient in [J/m^2^K], *k* is the thermal conductivity of the fluid in [J/mK], D is a characteristic length in [m] (can be L in the case of flat plates, D if the flow is around a curved body), Re is the Reynolds number [dimensionless] and Pr is Prandtl number [dimensionless], C, m and n are coefficients that correspond to the original data fit and are available in all heat transfer textbooks.

Eq. (2) has two parts and is solved for Nu from a fluid flow perspective first. This part involves evaluating Re as well as selecting a value for Prandtl number, which relates momentum diffusivity to thermal diffusivity (which is almost fixed for specific fluids and available in literature). Then, the convective heat transfer coefficient can be estimated by multiplying the freshly found Nusselt number with the thermal conductivity of the fluid and dividing it by the characteristic length. The value of (*h*) can then be substituted into Eq. (1) and the heat transfer is estimated with the pertinent accuracy limitation that might necessitate quoting the value with a higher and a lower limit and make the design decision accordingly.

The above procedure, with its limitations and implications might be clear for all scholars working with heat transfer. In order to carry out such a standardized test, there are different aspects that should be taken into account, including sample preparation with uniform dispersion and a stable homogeneous nanofluid. Moreover, preventative measures must be upheld to avoid agglomeration and precipitation. Furthermore, characterization techniques that take into account the thermal conductivity of both nanoparticles and nanofluids, viscosity measurements, particle size distribution, and the Zeta potential which studies the dispersion of nanoparticles within nanofluids with time, must be conducted. There is also a need for standardized nanofluids to measure the performance of rising nanofluid combinations upon. Additionally, measurement conditions including temperature, flow velocity, viscosity, and density should be reported in order to allow the reproduction of reported results, all while taking into account error, statistical, and uncertainty analysis.

What this work found in most of the literature surveyed, however, is that only a small percentage of authors went through the exercise of comprehensively assessing the convective heat transfer problem from both fluid flow and thermophysical properties perspectives. The discretization of the naturally continuous and interconnected problem of convective heat transfer is the trap that most researchers appear to have fallen into. As the readers will later see, most authors measured the thermophysical properties of the nanomaterial to be added to the fluid in a certain proportion, adjusted these properties for the nanofluid as an arithmetic average of the amount of fluid times its properties, with the amount of nanomaterial times its properties, and plugged these numbers into deterministic heat transfer equations, which provided a seemingly impressive lines conforming on one another. This is the first major source of bias that will be highlighted in this paper.

The other source of bias that will be discussed in this work is the experimental setups for heat transfer measurement. Most of the setups have thermocouples and thermometers that measures temperature change and plots the performance of the nanofluid at different concentrations of nanomaterial in the solvent. The thermophysical properties are also plotted, but the number of publications that carries out sensitivity analysis or a correct error analysis are also limited. So far, and as will be presented later, the data show obvious overlap with baseline readings, where the whole enhancement would appear to be within the natural variation of the instruments and the process.

Finally, the promised enhancement that these nanofluids offer in heat transfer for heat exchangers will be examined from maintenance and implementation points of view. Most heat exchanging equipment are sensitive to nanoscale particles accumulation over their lifetime, as these particles cause malfunctions or can impede heat transfer. There is also the issue of the cost of such material, as many authors opt for gold and sometimes diamond nanoparticles that have limited chances of being recovered or recycled in the system without significant loss.

## Geographical distribution of publications

**Figure 1:**
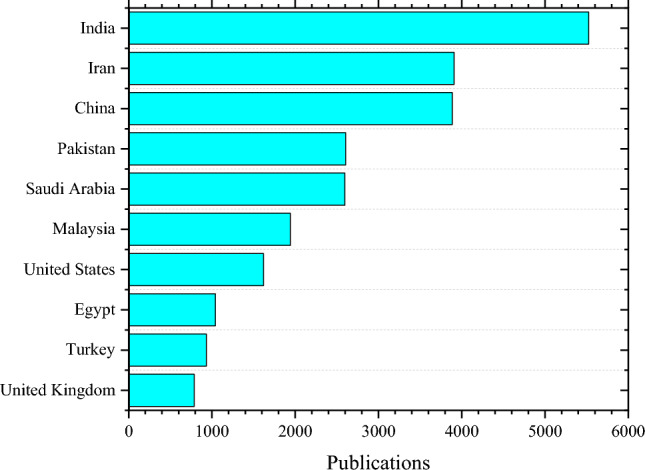
Publications on nanofluids by region between 2010 and 2022.

**Figure 2:**
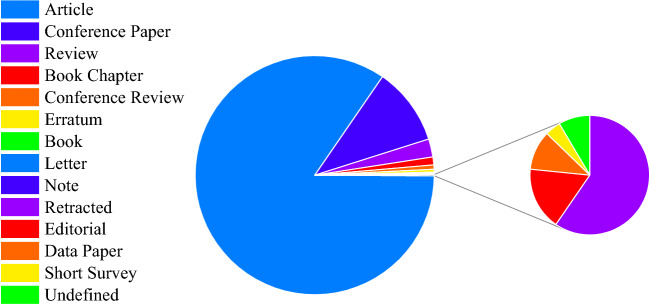
Classification of publications.

Research on nanofluids has increased dramatically in the last decade, where numerous experimental and theoretical investigations have been carried out on different aspects of nanofluids^6^. In this analysis, a Scopus database search was conducted to highlight scientific research papers published using the keyword “Nanofluids”. This search and subsequent analysis covers years 2010 to 2022, and shown in Fig. 1. A total of 124 countries and territories contributed articles to the scene. Where India ranked top in nanofluid publications with 5524 papers. Similarly, researchers in Iran and China published 3907 and 3885 papers, respectively. Furthermore, Pakistan, Saudi Arabia, Malaysia, the United States, Egypt, Turkey, and the United Kingdom also made notable contributions. In terms of publication types, Fig. 2. shows that the majority of these publications are journal articles, followed by conference papers, reviews, and others.

## Heat exchanger design

To be able to compare enhancement in heat transfer due to working fluid substitution, the American Society of Mechanical Engineers (ASME) standards should be invoked. These standards, coupled with engineering heat transfer textbooks single out the double-pipe counter flow heat exchanger as the De facto basis of comparison of different heat transfer changes attributed to the utilization of different working fluid. Usually, the basic run consists of pure water circulated into the heat exchanger using pumps, while temperature variations at inlets and exits of the cold side vs. hot side are recorded and compared. Different flow rates, and thus Re numbers, are also tried so that the temperature profile at steady state operation is then used to estimate Nu and consequently find an estimate to the convective heat transfer coefficient.

In general, there is a lack of consensus in literature as which heat exchanger geometry should be the standard for testing heat transfer enhancements from the addition of nanofluids. Although the simple double-pipe heat exchanger equipped with appropriate temperature, pressure and flow telemetry and data acquisition setup would be expected to standardize the outcome of such experiments and make any comparison more valid and valuable. The most common heat exchangers used for experiments are the circular tube, double pipe and shell-and-tube types. A summary of each is given in the following sections, along with reflections from the authors on the significance of claimed heat transfer enhancement in cited literature.

### Circular tube heat exchanger

Convective heat transfer through circular tubes is vital to investigate, due to the multitude of applications that depend on the heat transfer characteristics of fluids inside such tubes. Circular tubes are employed in heat exchangers, boilers, cooling, and solar thermal techniques such as parabolic trough collectors. The application of nanofluids to improve the heat characteristics of fluids inside circular tubes has been extensively studied both experimentally^7–9^ and analytically^10,11^. The convective heat transfer coefficient of the fluid inside the circular tube is calculated using the following equation:

3 $$\mathrm{h}=\frac{\mathrm{Nu}\cdot \mathrm{k}}{\mathrm{D}}$$

where the Nusselt number depends on various variables such as the flow type, boundary conditions, Reynolds and Prandtl numbers. For instance, the Nusselt number for a fully developed laminar flow in a circular tube with constant wall temperature be calculated using the following correlation:

4 $$\mathrm{Nu}=3.66 \; (\mathrm{Constant \; wall \; temperature},\mathrm{ laminar})$$

For fully developed laminar flow in a circular tube with constant wall heat flux, the Nusselt number can be calculated using the following correlation:

5 $$\mathrm{Nu}=4.36\;(\mathrm{Constant \; wall \; heat \; flux},\mathrm{ laminar})$$

For turbulent flow regimes in a circular tube with constant wall temperature, the Nusselt number can be calculated using the following correlation known as the Dittus Boelter correlation:

6 $$\mathrm{Nu}=0.023{\mathrm{Re}}^{0.8}{\mathrm{Pr}}^{0.3}$$

For turbulent flow regimes in a circular tube with constant wall heat flux, the Nusselt number can be calculated using the following correlation:

7 $$\mathrm{Nu}=0.0296{\mathrm{Re}}^{0.8}{\mathrm{Pr}}^{0.3}$$

**Figure 3:**
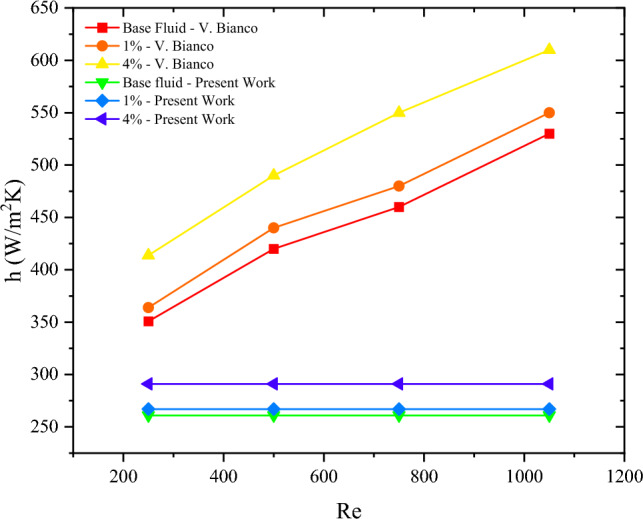
Comparison between present work calculations and Bianco^12^ for the heat transfer coefficient.

Bianco et al.^12^ numerically investigated the heat characteristic of a fully developed laminar flow water-Al_2_O_3_ nanofluid in a circular tube. The variation of the Reynolds number and the volume fraction on the convective heat transfer was also studied. The Reynolds number varied between 250, 500, 750, and 1050 respectively. While volume fractions of 1% and 4% were employed. The numerical results showed an improvement of 14% in the convective heat transfer coefficient for a volume fraction of 4% and a Reynolds number of 250. Nevertheless, by employing the same parameters from the study and calculating the improvement in the heat transfer coefficient through the above correlations, an improvement of merely 10% is recorded. This showcases that the calculation of the convective heat transfer coefficient through suitable Nusselt number correlations yields less enhancement, also shown in Fig. 3^12^.

Ali^10^ experimentally investigated the convective heat transfer of SiO_2_/water turbulent flow inside a copper circular tube. The volumetric concentrations of the SiO_2_ particles was varied between 0.001%, 0.003%, and 0.007%. The experimental results reported an increase in heat transfer coefficient of approximately 27% when using SiO_2_ nanoparticles at a concentration of 0.007%, compared to deionized water, at the highest Reynolds number of 19,500. At a lower concentration of 0.001% SiO_2_ nanoparticles, the maximum enhancement in heat transfer was observed to be around 8–9%. As the concentration of SiO_2_ nanoparticles increased, there was an observed increase in convective heat transfer coefficient. Nevertheless, by utilizing the above correlations and calculating the heat transfer coefficient through the Nusselt number, an increase of only 2.94% and 3.44% was recorded for the heat transfer coefficient of the nanofluids at concentrations of 0.007% and 0.001%, respectively. Table 2 shows a comparison between the obtained results from^10^ and the calculated results.

**Table 2:** A comparison between the obtained results from^10^ and the calculated results.

Parameters	Published work^10^	Present work
Nanofluid	SiO_2_/water	
Reynolds number	19,500	
Heat transfer coefficient enhancement (0.001%)	8–9%	2.94%
Heat transfer coefficient enhancement (0.007%)	27%	3.44%

Karabulut et al.^9^ carried out a numerical and experimental analysis to study the convective heart transfer of graphene oxide (GO)/ water nanofluid under turbulent flow inside a circular tube. The convective heat transfer coefficient was obtained through numerical analysis, then utilized to calculate the value of the Nusselt number. The results obtained in this study can be summarized as follows: The average increase in the convection heat transfer coefficient was 29% when using a nanofluid with 0.01 vol% GO concentration, at a Reynolds number of 5032. However, when the concentration of GO-DW was increased to 0.02 vol%, the enhancement in the convection heat transfer coefficient reached 48%. Once again, calculating the convective heat transfer based on the above correlations and utilizing the same exact parameters, yielded that the presence of nanoparticles adversely affected the heat transfer characteristics. For instance, the addition of 0.01% GO reduced the convective heat transfer by 5% and the addition of 0.02% reduced it by 4%. Table 3 shows a comparison between the obtained results from^9^ and the calculated results.

**Table 3:** A comparison between the obtained results from^9^ and the calculated results.

Parameters	Published work^9^	Present work
Nanofluid	GO/water	
Reynolds number	5032	
Heat transfer coefficient enhancement (0.001%)	29%	− 5%
Heat transfer coefficient enhancement (0.007%)	48%	− 4%

### Double pipe heat exchanger

Concentric tube heat exchangers are widely utilized across various applications, serving purposes such as air-conditioning, oil cooling, refrigeration, engine cooling, and material processing. As a result, concentric tube heat exchangers can be used in several industries, including chemical, refinery, and pharmaceutical industries. The widespread use of concentric tube heat exchangers is due to their low cost, high reliability^13^, simple design, and robust configuration^14^. A concentric tube heat exchanger generates a temperature gradient by employing various fluid streams at unique temperatures in parallel, divided by a pipe. This, in return, causes forced convection, resulting in heat transfer^15^. However, there are several disadvantages accompanied with concentric tube heat exchangers. For instance, a concentric tube heat exchanger can lose heat due to its large outer shells. As a result, various researchers attempted to enhance the heat transfer properties of a concentric heat exchanger via different methods such as induced surface vibrations, and air bubbles injection^16,17^. The most predominant method is by using nanofluids^18^.

Several researchers reported that the using nanofluids in concentric tube heat exchangers can enhance thermal conductivity, Nusselt number, and convective heat transfer properties of the nanofluid^19–21^. For instance, Akyürek et al.^22^ investigated the heat transfer and pressure drop characteristics of Al_2_O_3_-Water nanofluids in a concentric tube heat exchanger with and without wire coil turbulators. The investigation showed that Nusselt number increased with an increase in the particle concentration and Reynolds number, leading to an enhancement in the heat transfer coefficient. The authors used Gnielinski’s equations for computing the Nu and friction factor to validate their experimental results. Equations (8) and (9) demonstrate Gnielinski’s equations for the Nusselt number and friction factor, respectively^23^. The reported Nusselt number associated with the 1.6% Al_2_O_3_-Water nanofluid at a Re number of 20,000 is between 350 and 400. However, based on the correlations presented in Eqs. (10) and (11), the calculated Nusselt number is approximately 170.93. This Nusselt number is computed based on friction factor of 0.0261, Re number of 20,000, volume fraction of 1.6%, and Pr of 10.07669. The Pr number is acquired based on Eq. (12). The significant difference between these Nusselt numbers suggests that the enhancement of the Nusselt number is far less than it is mentioned.

8 $$Nu=\frac{(\frac{f}{2})(Re-1000)Pr}{\left(1+12.7\left(\frac{f}{2}^{0.5}\left({Pr}^\frac{2}{3}-1\right)\right)\right)}$$

9 $$f={((1.58\times \mathrm{ln}\left(Re\right))-3.82)}^{-2}$$

10 $$Nu=\frac{(\frac{f}{8})(Re-1000)Pr}{\left(1+12.7\left({\frac{f}{8}}^{0.5}\left({Pr}^\frac{2}{3}-1\right)\right)\right)}$$

11 $$f={((0.79\times \mathrm{ln}\left(Re\right))-1.64)}^{-2}$$

12 $$Pr=\frac{{Cp}_{nf}\times {M}_{nf}}{{K}_{nf}}$$

where *Cp*_nf_ is the specific heat of the nanofluid, *M*_nf_ is the viscosity of the nanofluid, and *K*_nf_ is the thermal conductivity of the nanofluid.

In the same way, Sonawane et al.^18^ investigated the heat transfer properties of Al_2_O_3_-Water nanofluids in a copper concentric tube heat exchanger. The authors compared the heat transfer of the nanofluid to the base fluid (water). The study showed that the nanofluids exhibited higher heat transfer rates as the concentration of the nanofluid increased. Nonetheless, the authors claimed that the Nusselt number of the nanofluid at 3% concentration is between 15 and 16; whereas, after calculating the Nusselt number by the correlation demonstrated in Eq. (6), the Nusselt number is around 26.16. The Nusselt number was computed after setting the Re number to 4000 and Pr value found to be around 4.061 and the friction factor is approximately 0.0414. Similarly, Khalifa and Banwan^24^ studied the increase in the heat transfer rate after adding *y*-Al_2_O_3_ nanoparticles to water in a concentric tube heat exchanger. The authors reported that the enhancement in the convective heat transfer increased as the nanoparticle volume fraction and flow rate increased. Furthermore, the authors attained a maximum enhancement of 20% in the Nusselt number and 22.8% in the heat transfer coefficient at 1% volume fraction and 6026 Re number. In order to validate the accuracy of the Nusselt number and heat transfer coefficient, the authors compared these values to the values estimated based on empirical correlations. The Dittus-Boelter correlation, as shown in Eq. (6), was employed to estimate the Nusselt number. The values from the experimental setup and from the Dittus-Boelter correlation were highly correlated. However, the Dittus-Boelter correlation is only used under the condition that the Re number is higher than 10,000. Nonetheless, the highest Re number in this study is 6026, indicating that this correlation might compute inaccurate results. Through this correlation, the Nusselt number at 1% volume fraction and at 6026 Re number is between 55 and 65. Nevertheless, the correlation presented in Eq. (10) showed a different Nusselt number from the one computed by the Dittus-Boelter correlation. The correlation showed that the Nusselt number is around 41.261, the friction factor is 0.0364, and the Pr number is 4.4071. The computed Nusselt number is much lower than that reported by the authors, suggesting a lower enhancement in the heat transfer coefficient.

### Shell and tube heat exchangers

Due to its small size and high heat transfer rate, the shell and tube design is the most commonly employed design in heat exchangers. In general, to increase the heat transfer rate through heat exchangers, fluids with high heat transfer coefficients are used^25^. In this section, the heat transfer coefficient of shell and tube heat exchangers applying different nanofluids are investigated and compared with other correlations from literature. Said et al.^26^, examined the heat transfer characteristics of a CuO/water nanofluid mixture. The nanoparticle concentrations employed were 0.05, 0.1, and 0.3 vol%. Twenty eight carbon steel tubes and one carbon steel shell were used for the experimental setup. CuO/water was circulated within the heat exchanger's tube section. In addition to the experimental investigation, a theoretical model was created to verify the outcomes. The findings demonstrated that for the same fluid inlet temperatures and mass flow rates, the convective heat transfer coefficient obtained when using the proposed nanofluid is more than when using the basefluid. The heat transfer enhancement achieved was 7%. The input and output data were obtained and then validated as shown in Table 4 using the implemented correlation. Nusselt number was calculated using the correlation in Eq. (13) for concentrations up to 2 vol% and for turbulent flow:

13 $${\mathrm{Nu}}_{\mathrm{nf}} = 0.0059(1+7.6286{\mathrm{d_{\mathrm{p}}}}^{0.6886}{\mathrm{Pe}}_{\mathrm{nf}}^{0.0010} ) ({\mathrm{Re}}_{\mathrm{nf}}^{0.9238} {\mathrm{Pr}}_{\mathrm{nf}}^{0.4})$$

where dp is the nanoparticle diameter (50 nm).

**Table 4:** Data obtained using CuO/water nanofluid.

Volume fraction (%)	0.05
Re	2628.875279
Pr	6.66995
D (m)	0.0055372
Viscosity (kg/m s)	0.00105315
Density (kg/m^3^)	1000
Velocity (m/s)	0.5
Specific heat (J/kg K)	4180
k (W/m K)	0.66
α	2.7588
Pe	9.06191 × 10^–9^
dp (m)	0.00000005
Nu	18.18507429
h (W/m^2^ K)	2167.548406

The heat transfer coefficient is then calculated based on the Nusselt number from Eq. (13):

The results were validated after getting close values of Nu and h to the values reported in the paper which are 18.9 and 2255.39 (W/m^2^ K) respectively. Hence, proving the enhancement in the heat transfer coefficient compared to water with h value equal to 1998.47 W/m^2^ K.

Similarly, Ghozatloo et al.^27^ investigated the heat transfer coefficients of water-based graphene nanofluids in the point of entry as well as in laminar conditions using a shell and tube heat exchanger. A flowrate of 0.8 L/min was used for establishing the laminar flow. Based on their findings, introducing 0.075% graphene to the base fluid improved the thermal conductivity up to 31.83% at saturation concentrations of graphene and improved the heat transfer coefficient depending on the conditions of flow. At 38 °C, the convective heat transfer coefficient of graphene nanofluids increased by 35.6% in comparison with purified water using an amount of 0.1 wt%. In the conducted analysis, the local heat transfer coefficient was determined based on the fluid temperature in *x*_*i*_ section (*T*_*fi*_) and test part inner wall temperature (*T*_*wi*_). Then, *h* was calculated from:

14 $$h= \left(\frac{{q}^{\prime\prime}}{{T}_{wi}-{T}_{fi}}\right)$$

where q″ is the constant heat flux of 5429 W/m^2^. Then, the average heat transfer coefficient was calculated by taking the arithmetic mean of the obtained local heat transfer coefficients.

In our analysis, Shah’s correlation for laminar flow was used to validate the Nusselt number as shown in Eq. (15)^28^. It is also important to note that the analysis was conducted for the different volume concentration of graphene used.

15 $$Nu = 1.953 {\left(RePr\frac{{d}_{i}}{L}\right)}^\frac{1}{3}, \; for \; \left(RePr\frac{{d}_{i}}{L}\right) \ge 33.3$$

where L is the length of the horizontal circular copper tube used.

Then, the heat transfer coefficient is calculated based on the Nusselt number from Eq. (13). In Table 5, the average heat transfer coefficients, which depend on the average temperature, are shown which indicate notable variations. In the conducted analysis, by increasing the temperature and concentration of graphene nanoparticles, the average heat transfer coefficient increases. However, in the results obtained in Table 6 after calculation based on the correlations, a different trend is found where the heat transfer coefficient was increasing to the threshold of 0.048vol% in KRG-4 sample where it decreased. Table 6 shows the calculated heat transfer coefficient using water and nanofluids.

**Table 5:** Average heat transfer coefficient using water and nanofluids from the reference^27^.

T_avg_ (°C)	Water	Average heat transfer coefficient (W/m^2^ K)		
		KRG-2	KRG-3	KRG-4
25	1585.7	1700.3	1816.0	1942.3
32	1692.1	1867.5	2017.7	2183.2
38	1882.2	2102.6	2329.1	2553.1

**Table 6:** The calculated heat transfer coefficient using water and nanofluids.

Water	Heat transfer coefficient (W/m^2^ K)		
	KRG-2	KRG-3	KRG-4
311.881	348.3228	377.317	344.8885

Farajollahi et al.^29^ examined the heat transfer properties of γ-Al_2_O_3_/water and TiO_2_/water nanofluids in a shell and tube heat exchanger with turbulent flow conditions. The effects of Peclet number, volume concentration of suspended nanoparticles, and particle type on heat transfer were studied. Data related to the velocity and flow rates were missing, hence, the velocity was calculated based on the Peclet number as shown in Eq. (16) in order to obtain other parameters from Eqs. (3) and (13).

16 $$V= \frac{Pe \times \mathrm{ \alpha }}{\mathrm{d_{\mathrm{p}}}}$$

Furthermore, the viscosity of the nanofluid was calculated using Eq. (17):

17 $${\mu }_{nf}=\left(1+\frac{5\phi }{2}\right){\mu }_{w}$$

where *ϕ* is the volume concentration of the nanoparticles.

**Figure 4:**
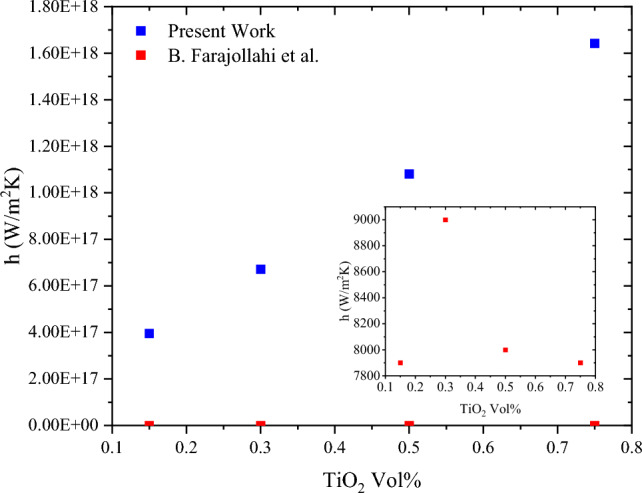
Comparison between present work calculations of the heat transfer coefficient and Farajollahi et al.^29^.

The calculations were implemented using the different volume percentages of TiO_2_ and using the obtained Peclet number. In their work, the correlation in Eq. (13) was used to calculate the Nu before determining the heat transfer coefficient. A significant difference is found between both results, using 0.15%, 0.3%, 0.5% and 0.75% of TiO_2,_ as shown in Fig. 4.

Anitha et al.^30^ reported the testing of three types of nanofluids, namely Al_2_O_3_-Cu-Water, Cu-Water, and Al_2_O_3_-Water for different volume concentrations of 1%, 2%, 4%, 8%, 10%, and 20%. According to the authors, the Reynold’s number that was used for the analysis was equal to 844.4, which falls into the laminar flow region.

For a circular tube, which is the case for the tube in a shell-and-tube heat exchanger, that harbors a laminar flow, the Nusselt number is independent of the Reynold’s and Prandtl numbers for a constant heat flux, bearing a constant value of 4.36 as shown in Eq. (5)^31^.

In their work, the authors calculated the heat transfer coefficient using Eq. (14):

With a diameter of 0.033 m, the only thing left that can have a significant impact on the heat transfer coefficient is the value of the thermal conductivity of each corresponding nanofluid, which does not change much with changing the concentration. The value of the heat transfer coefficient in the present work is calculated using the following equation:

18 $$h=Nu\times \frac{k}{D}=4.36\times \frac{k}{0.033}$$

However, there is barely any significant variation of the thermal conductivity values, that according to Eq. (18) ultimately dictate any changes in the values of the heat transfer coefficient. Which is why in this present work, the values of the heat transfer coefficient were recalculated using the aforementioned Nusselt correlation in Eq. (18).

**Figure 5:**
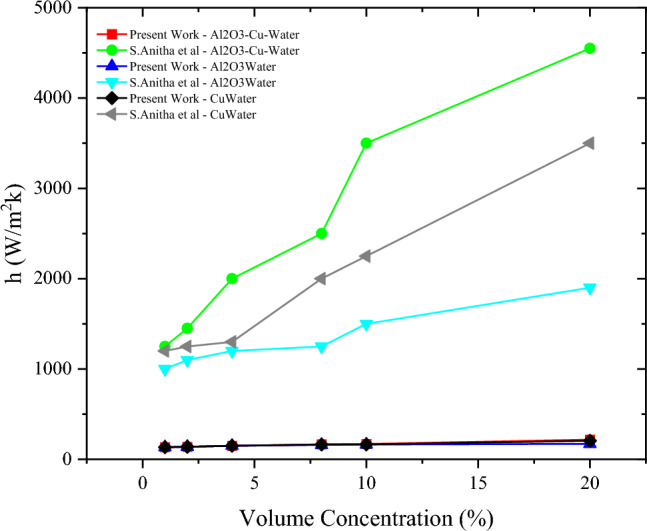
Comparison between heat transfer coefficient values recalculated in the present work and those found in Anitha et al.^30^.

The values presented by Anitha et al. are at least 10 times more than the values obtained through the suitable Nusselt correlation for laminar flow in circular tube with a constant heat flux. Figure 5 is a visual representation of that undeniable difference. The values calculated in the present work indicate that there is no enhancement in the heat transfer coefficient associated with the addition of nanoparticles, no matter the vol%, contrary to those shown by Anitha et al.

In a similar work conducted by Kuman and Sonawane^32^, they tested the effect of enhancing the heat transfer coefficient by adding Fe_2_O_3_ nanoparticles to water and ethylene glycol (EG), in the tube of a shell-and-tube heat exchanger. Throughout the conducted tests, they maintained the hot stream and two constant temperatures 50 $^\circ$C and 80 $^\circ$C, while ranging the vol% of the nanoparticles from 0.01 to 0.08. The reported thermal conductivity values ranged from 0.614 to 0.651 W/mK and 0.252 to 0.296 W/mK with changing the vol% from 0.01 to 0.08 for Fe_2_O_3_-water and EG, respectively. For a constant hot stream temperature operation, the Nusselt number is constant in the laminar flow region, similar to the case mentioned earlier, however having a different value as shown in Eq. (4)

With a tube diameter of 0.0107 m, the heat transfer coefficient is only a function of the thermal conductivity and is obtained through the following equation.

19 $$h=\frac{3.66\times k}{0.0107}$$

However, the authors used Eq. (14) to obtain h, and then used those values of h to obtain values for Nu.

**Figure 6:**
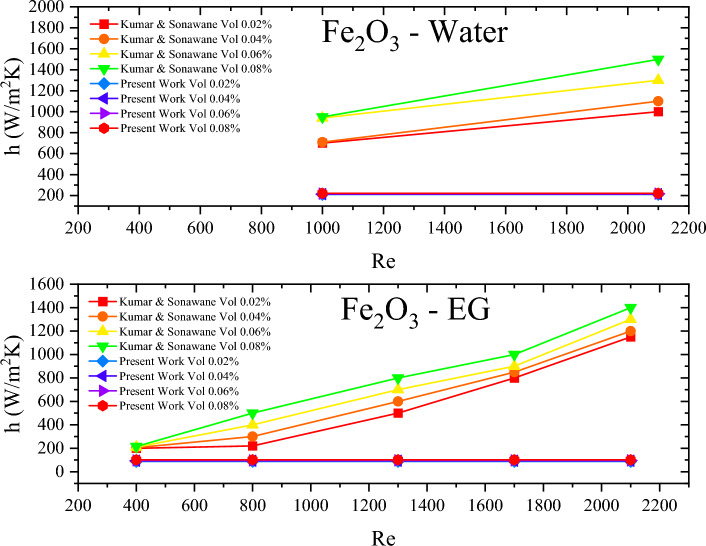
Comparison between heat transfer coefficient values recalculated in the present work and those found in Kumar and Sonawane^32^.

The base comparison between the present work and that conducted by Kumar and Sonawane will be confined to the laminar region of their reported work, which is presented in Fig. 6. Moreover, throughout their tests, they did not exceed a Re of 10,000, all while using correlations for Nu comparison that are only valid at Re > 10,000, such as the Dittus-Boelter and the Gnielinksi correlation, which is invalid.

## Challenges and limitations of nanofluids

### Microstructure-imposed challenges

There are various challenges that are imposed solely by the microstructure of nanoparticles which are revealed by an analysis of the existing scientific literature^33^. A common issue is regarding the uniform dispersion of nanoparticles within the base fluid. The use of Scanning Electron Microscopy (SEM) by researchers often reveals clumped regions of nanoparticles which raises concerns about the reliability and effectiveness of such mixtures and enhancements.

**Figure 7:**
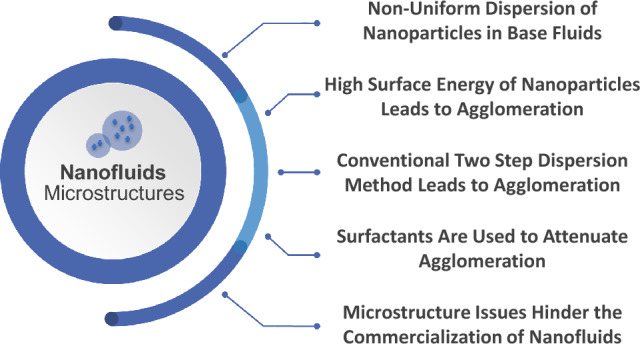
Summary of microstructure issues in nanofluids.

Figure 7 presents the current causes and challenges imposed by the microstructure of nanofluids.

Uniform dispersion of nanoparticles in a base fluid is a core condition for ensuring optimal heat transfer performance of nanofluids. Despite extensive efforts by researchers in the surveyed literature, achieving a homogeneous dispersion remains a significant challenge. This is traced back to various factors that collectively contribute to this phenomenon, including high surface energy of nanoparticles that lead to a natural tendency to form clusters and agglomerate. Strong forces such as Van Der Waals attractions, can, and in most cases do, overcome the repulsive forces between nanoparticles^34^. The formation of agglomerates can be seen in following works^35–39^. Because it is more economical and straightforward, producing nanofluid by dispersing nanoparticles in base fluid using the two-step method has become prevalent on a large scale, which ultimately affects the commercialization of nanofluids. The main disadvantage of this approach is agglomeration caused by Van Der Waals forces and the cohesive strength of the individual nanoparticles^40^. This continues to be a concern in the production and use of nanofluids because this behavior has an effect on the entire fluid stability. Fluid flow properties in porous media, such as viscosity, in addition to cooling applications, can be restricted by such agglomeration^41^. Sedimentation, abrasion and reduced nanofluid enhancement effects are the negative impacts that may occur as a result of agglomeration^42^.

Moreover, the interaction between the nanoparticles and the base fluid extremely influences the dispersion behavior. The addition of surface modifiers and surfactants is often done to improve the compatibility between the base fluid and nanoparticles^43^. However, despite these efforts, nanoparticles may still experience poor dispersion due to confined interactions with the base fluid, as can be seen in most published literature. Properties, such as the base fluid viscosity and surface tension, play a crucial role in governing these interactions and consequently the nanoparticles dispersion^44^ and are claimed to have an effect on heat transfer. More importantly, the non-uniform dispersion of nanoparticles in base fluids poses significant challenges when attempting to elevate these nanofluids from waivered laboratory findings to real-world applications.

### Commercialization and scaling-up challenges

There are two main challenges that hinder the commercialization and utilization of nanofluids in large-scale heat exchangers. First, potential adverse physical effects of the solid matter on the internal workings of candidate systems. Nanoparticles used in experiments in available literature are strikingly similar to materials that cause internal fouling of heat exchanger pipes. Given the uncertainty in calculating heat transfer enhancements achieved exclusively because of the addition of nanoparticles, as well as the significantly higher heat transfer enhancement that result from increasing the working fluid flow rate (at constant heat input), there is a lack of commercially available products on the market that utilize nanofluids. There have been 24 patents involving devices and technologies that employ nanofluids since 2001^45^ from solar collectors to microchip cooling applications, which impose application-specific challenges, but so far there exists no market penetration for nanofluid based devices.

**Figure 8:**
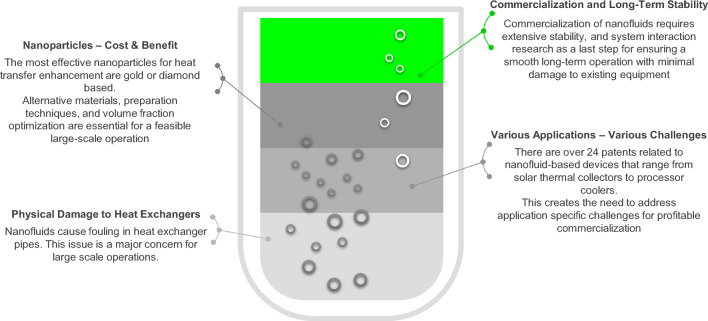
Steps and measures for nanofluids commercialization.

The second reason for product availability is the price of nanoparticles. The most effective nanoparticles reported in literature are either gold, diamond, or silver, which are estimated to cost around 80$/g, 35$/g, and 6$/g, respectively. The volume fraction of these particles and their non-recyclability preclude any enthusiasm in employing them on a wide scale. The high prices of nanofluids are due to numerous factors, involving material costs, preparation costs (which include reagent, surfactant, ultrasound bath, and stirrers), and labor costs. Also, the documented techniques for synthesis are frequently modified and differ from one application to another. As a result, there is inadequate knowledge and data to properly determine the price of nanofluids, which adds another obstacle^46^. Moreover, the stability of nanoparticles in nanofluids is a gray area in that specific field of research. Researchers apply what is known as the Zeta potential test in order to test for the stable dispersion of nanoparticles and their resistance to precipitation. However, practical long-term stability of nanofluids has yet to be tested. Additionally, long term effects of nanofluids, whether on human health or environmental toxicity, are still vague. Despite the proclaimed advancements in nanofluids research there have yet to be comprehensive studies regarding the long-term exposure and interactions of nanofluids within targeted systems. Furthermore, nanofluids should be assessed for their capability to retain their required characteristics over operating conditions (e.g., thermal conductivity following heating and cooling cycles). The development of nanofluids based on water with the required improved thermal and mechanical properties is still a challenge^42^. Due to that, there has yet to be a breakthrough in mainstream markets where most consumers reside. Solid work has to be laid out which harbors stability and efficiency, to convince industries and end consumers to adopt nanofluids as a replacement for well-established heat transfer fluid alternatives. A summary of the steps and measures required, as well as the challenges to be addressed for nanofluids' commercialization is shown in Fig. 8.

## Conclusion

This study presented an overview of the seemingly hot topic of nanofluid utilization for heat transfer enhancement. In particular, issues pertinent to a consensus-by-volition in calculating heat transfer coefficients, h, in nanofluids research. Many studies rely on simplistic equations, such as q/$\Delta$ T, which overshadows the undeniable complexity of convective heat transfer phenomena. Even in studies that utilize empirical formulae that involve the Nusselt number, Nu, many erroneous validation steps resulted from correlations made at invalid ranges of Reynolds numbers or depending solely on the minute changes in the thermal conductivity of the working fluid due to increased volume fraction (quantity) of nanofluid. Most of papers reporting on the latter have neatly plotted graphs due to said small changes in volume fraction that rarely relied on experimental rigor to determine the constants in the Nusselt equation. Furthermore, in laminar flow regimes, where Nu remains constant, it is essential to recognize that *h* is primarily a function of the thermal conductivity in that case, rather than Re, which does not significantly change with the addition of nanoparticles.

In its current form, the nanofluids research area requires a standard for independently evaluating the effect of nanoparticles addition to working fluids. The available results in literature indicate that increasing the flow rate within the heat exchanger could enhance the heat transfer at higher order of magnitudes compared to any enhancement gained from nanoparticle addition. And finally, the independence of the results is overshadowed with the specificity of the geographic region and repetition of certain lead figures in the field. A more critical and impartial standards must be set for a field that is growing as fast and vast as nanofluids.

## Data Availability

The datasets used and/or analyzed during the current study are available from the corresponding author on reasonable request.
